# Contemporary Review of Minimally Invasive Mitral Valve Surgery: Current Considerations and Innovations

**DOI:** 10.3390/jcdd11120404

**Published:** 2024-12-14

**Authors:** Sharifa Alsheebani, Daniel Goubran, Benoit de Varennes, Vincent Chan

**Affiliations:** 1 Department of Surgery, McGill University, Montreal, QC H3A 0G4, Canada; sharifa.alsheebani@mail.mcgill.ca (S.A.); benoit.devarennes.med@ssss.gouv.qc.ca (B.d.V.); 2 Department of Surgery, University of Ottawa Heart Institute, Ottawa, ON K1Y 4W7, Canada; dagoubran@ottawaheart.ca

**Keywords:** minimally invasive, mitral valve, mini-mitral surgery

## Abstract

Minimally invasive mitral valve surgery (MIMVS) has become a well-established alternative to traditional median sternotomy at high-volume surgical centers. Advancements in surgical instruments have led to further refinement of MIMVS. However, MIMVS remains limited to select patients in select settings. In this review, we provide a brief overview of the evolution of MIMVS, as well as a technical description of the most relevant aspects of minimally invasive mitral valve surgery.

## 1. Introduction

Minimally invasive mitral valve surgery (MIMVS) refers to a collection of operative techniques and surgical technologies performed via smaller incisions, aimed towards the avoidance of surgical chest trauma inflicted by standard median sternotomy [1,2]. Today’s interest and demand in MIMVS is largely driven by patient and provider desire to have a shorter recovery, reduced pain profile and better aesthetics, without sacrificing risk or quality of surgery, by the patient’s conviction that smaller incisions correlate with reduced pain, therefore allowing for overall faster postoperative recovery [3,4,5].

A particular challenge with mitral valve surgery is often optimizing exposure within a small deep space while allowing for an enface view of the mitral valve. Indeed, MIMVS, with the use of a thoracoscope and utilization of long-shafted instruments, may arguably allow for favorable exposure [3,6]. Unlike some other surgical subspecialties, the adaptation of MIMVS has long been challenged by skepticism, with the concern for the trade-off of incision size against the comfort and safety of standardized techniques with proven durable results. Despite such criticism, some institutions have adopted MIMVS and have published favorable results, both as observational and prospective studies [5,6]. Indeed, MIMVS appears to have favorable late outcomes both in terms of freedom from reoperation and survival compared to conventional methods [1]. Other reported benefits include improved postoperative respiratory function, lower rate of transfusions and the avoidance of sternotomy-related complications [3,7]. A shorter length of hospitalization and reduced time to return to normal activity were also highlighted [8].

One limitation for interpreting results relates to the heterogeneity of MIMVS. The spectrum of MIMVS approaches includes the following [3]:

￭ Partial sternotomy (upper and lower);
￭ Parasternal;
￭ Right anterolateral mini-thoracotomy without the use of a thoracoscope;
￭ Right anterolateral mini-thoracotomy with thoracoscopic assistance, including the use of a surgical robot;
￭ Port access with our without endo-aortic occlusion balloon;
￭ Percutaneous or trans-cutaneous approaches without cardiopulmonary bypass.

Each technique carries distinct advantages and limitations when compared to classic sternotomy, as well as when compared to one another [8]. A specially designed set of surgical instruments is required, along with careful collaborative surgical planning. The current paper will review the surgical techniques of MIMVS.

## 2. History and Evolution of MIMVS

When considering the application of MIMVS, the needs of the patient, along with their habitus and mitral pathology, are important considerations. Technical considerations include an approach to cardiopulmonary bypass (CPB) and myocardial protection [3]. The development of specialized instrumentation has allowed the gradual safe adaptation of such procedures over the years [9,10]. The first description of MIMVS was in the mid-1990s [1,6], with some of the initially proposed approaches still being used to some extent today. The right parasternal and transsternal incisions were described by Cosgrove and Cohen, with or without resection of the third or fourth costal cartilage [11]. This approach was soon abandoned, as its disadvantages included, but were not limited to, occasional chest wall instability, lung herniation, ligation of the right internal thoracic artery and difficult conversion to full sternotomy [11,12]. Subsequently, partial upper and lower sternotomies were later adopted by Cosgrove and Cohen, respectively [4]. Carpentier et al. pioneered the mini-thoracotomy video-assisted MIMVS approach in 1996, where he performed a mitral valve repair under fibrillatory arrest without aortic occlusion [1,11]. A major leap in the evolution of endoscopic mitral surgery was the development of three-dimensional (3D) vision and computer-assisted telemanipulation, allowing Carpentier et al. to also perform the first completely robotic mitral valve repair using the Da Vinci Surgical System (Intuitive Surgical, Sunnyvale, CA, USA) in 1998 [2,6,11,13].

## 3. Patient Selection Criteria

Not all patients are candidates for MIMVS; however, patient selection may also vary based on the center and treatment team. Complications may arise from an improper selection process. Some considerations are included below, although these may vary depending on the expertise of the treatment team [3,7,13,14,15,16,17,18,19,20,21,22]: 

Mitral Valve Pathology

￭ Severe mitral annular calcification (MAC) may require extensive annular decalcification and possibly patch reconstruction, although hybrid surgery can also be entertained;
￭ Mitral valve endocarditis may be associated with a peri-annular abscess, which may require debridement;
￭ Coronary artery disease in need of concomitant bypass.

Access Concern

￭ Prior right chest instrumentation or radiation. Increased risk of pleural adhesions may preclude safe dissection. In the case of prior cardiac surgery, meticulous dissection may allow for minimally invasive surgery.
￭ The size and shape of the patient can prevent achieving the desired access and exposure (obese patients and significant chest wall deformities).
￭ Inability to establish peripheral femoral cannulation (severe peripheral arterial disease, morbid obesity). To mitigate these limitations, some have advocated direct aortic or alternative arterial cannulation at the time of minimally invasive surgery.

CPB-related

￭ Challenging aortic cross-clamping and/or administering antegrade cardioplegia: prominent ascending aortic calcifications or aneurysm, moderate to severe aortic regurgitation.
￭ Conditions preventing efficient retrograde CPB perfusion via femoral cannulation: descending aorta aneurysm/dissection, aortic thrombus and size of peripheral vessels.

Cardiac Function

￭ Severe uni- or bi-ventricular dysfunction.
￭ Fixed severe pulmonary hypertension > 60 mmHg.
￭ Select untreated congenital anomalies.

Concomitant cardiac procedures such as ablation or right-sided interventions, tumor resection and atrial septal defect closure can easily be performed via MIMVS [14]. Additionally, patients who are deemed “too high risk”, mainly due to respiratory or renal problems, are a particular subgroup that could potentially benefit from minimally invasive approaches [3]. Importantly, end-organ dysfunction may also be a contraindication for MIMIVS. Patient selection, based on the comfort and experience of the team, is therefore paramount.

Redo operations should be carefully considered. In experienced hands, MIMVS may be useful in preventing damage to cardiac structures by avoiding prior surgical adhesions and allowing the preservation of coronary grafts [3,5]. However, in the event patient safety or surgical control might be jeopardized, an open approach is preferred. A classic sternotomy should also be used in patients with significant comorbidities or reduced ventricular function who would not tolerate a prolonged CBP to optimize both myocardial protection and operative time [3].

## 4. Preoperative Imaging Features

All patients being considered for MIMVS require preoperative imaging. A formal assessment based on echocardiographic and contrast-enhanced computed tomography (CT) features helps guide the selection process [3,6,15]. Similarly to the standard approach, transesophageal echocardiogram (TEE) verifies cardiac and valvular functions, while additionally allowing us to assess and guide access and cannulation techniques specific to MIMVS. In times of uncertainty between approaches, a decision can be deferred until the TEE is performed on the table.

Echocardiographic features that can make MIMVS challenging [3,6,15]:

￭ Extensive MAC.
￭ Moderate or greater aortic insufficiency.
￭ Left ventricular dysfunction.
￭ Severe pulmonary hypertension.

Understanding the patient’s thoracic anatomy is key to incision and port placement. A contrast-enhanced CT scan of the chest, abdomen and pelvis provides information on intercostal access, possible cannulation sites and perfusion strategies.

CT features that can make MIMVS challenging [3,6,15]:

￭ Suboptimal peripheral access quality (severe aortoiliac atherosclerotic disease, small femoral artery diameter, tortuosity) increases the risk of cerebrovascular events.
￭ Aberrant vasculature (discontinuous inferior vena cava, persistent left superior vena cava, or a retroesophageal left subclavian artery).
￭ Chest wall deformities, particularly pectus excavatum, can limit exposure due to the inability to retract the interatrial septum to properly expose the valve.
￭ Elevated right diaphragm.

## 5. Operative Set Up

### 5.1. Positioning and Port Placement

A right anterolateral mini-thoracotomy approach has become the most widely used technique for MIMVS. Patients are typically positioned supine, with a slight left-sided tilt via a roll or inflatable device under the patient’s right shoulder blade [3,6]. Defibrillator pads in place [6]. Groins prepared for femoral access (Supplemental Figure S1).

A 3–5 cm right-sided anterolateral incision is made, usually inferior to the nipple in men and within the inframammary crease in women [13]. The thorax is entered via the fourth intercostal space [13]. A soft tissue retractor with or without a rib spreader is utilized [3,6]. A range of additional incisions and ports are introduced depending on surgeons’ preferences and CPB strategy. In the event of encountering a high hemidiaphragm as anticipated from the CT, a retraction suture is placed in the central tendon of the liver and pulled to retract inferiorly through a separate stab incision [15]. Through the mini-thoracotomy incision, the pericardium is incised away from the phrenic nerve, extending superiorly to expose the ascending aorta and inferiorly towards the diaphragm [12,13,14]. Visualization may be enhanced using a thoracoscope, although some may elect to perform the operation under direct vision. In the case of robotic surgery, the arms must be positioned to avoid conflict with other instruments.

The advent of the surgical robot has made it feasible for port-access surgery to be performed. The advantages of robotically assisted mitral valve surgery include physical reach to the valve, which may be relevant in patients with a large body surface area, along with the benefits of visualization. However, favorable outcomes with robotically assisted mitral valve repair have been reported by dedicated treatment teams and specialized centers [23].

### 5.2. Establishment of CPB

Different sites of arterial and venous cannulation may be used, with peripheral cannulation being the most common. Arterial access is usually achieved with femoral artery cannulation [5]. The cannula is fixed to the skin to avoid displacement. Alternatively, the axillary artery or a central aortic cannula placed directly into the ascending aorta through a small incision over the second intercostal space can be attempted [3]. Venous drainage is typically achieved with a femoral multistage cannula advanced into the SVC under TEE guidance [3]. For some patients, such as those with a high BMI, a double femoral and internal jugular venous cannulation may be required to ensure adequate venous drainage [5]. A two-stage venous cannula may be placed directly into the right atrium through an incision in the fourth intercostal space [3,5]. Some may also employ mild hypothermia during the cardiopulmonary bypass run.

### 5.3. Aortic Cross-Clamping

Two common methods for aortic cross-clamping during MIMVS are currently used [3,11,12,21,22]:

￭ A direct transthoracic external, such as the Chitwood cross-clamp, may be passed through either the second or third intercostal space. Other novel commercially available aortic clamps include the Cygnet flexible clamp (Geister) and the detachable Glauber clamp (Cardiovision-Trytech, Tokyo, Japan)
￭ An internal endo-aortic balloon can be introduced in a retrograde fashion through the femoral artery and into the proximal ascending aorta under fluoroscopic or TEE guidance. This endo-balloon is then positioned just above the sino-tubular junction.

Advantages:

∘ Ability to deliver antegrade cardioplegia, vent the aortic root and monitor the root pressure through the tip of the endo-balloon.
∘ Advantageous when there is limited access to the aorta.

Disadvantages:

∘ Movement of the endo-balloon within the lumen may not be visible during the procedure. Bilateral axillary pressure monitoring is needed to detect displacement.
∘ Migration hazard into the arch (leading to neurological complications) or to the left ventricle (resulting in ventricular dysfunction)
∘ Aortic dissection is a feared complication.

Some surgeons report performing MIMVS on a beating vented heart without cross-clamping or inducing ventricular fibrillation [3,5]. While technically feasible, this approach increases the risk of air embolism or may be challenging for visualization should the patient have aortic insufficiency [5,12]. The risk can be reduced by flooding the operative field with carbon dioxide, having the patient in the Trendelenberg position and keeping the left ventricle empty and arterial pressure high on CPB [11,12].

### 5.4. Myocardial Protection

Both anterograde and retrograde cardioplegia may be used. When using an external clamp, antegrade cardioplegia can be delivered via direct catheter deployment into the aortic root. In the event an aortic endo-clamp is used instead, as described above, a cardioplegia solution can be delivered through the endo-clamp [10,12]. Retrograde cardioplegia can be delivered through either a directly placed coronary sinus cannula or, alternatively, through a percutaneous coronary sinus catheter placed by an aesthetist through the internal jugular vein [3]. Longer-acting cardioplegic solutions such as Del Nido or Custodiol^®^ cardioplegia can be considered [3,6].

### 5.5. Surgical Procedure: MV Repair or Replacement

Following the dissection of Sondergaard’s groove and left atriotomy, an atrial retractor is selected to display the mitral valve [15]. This retractor is often placed through a small incision in the fourth intercostal space [3]. As visualization is optimized, the mitral valve anatomy can be properly assessed. A variety of instruments have been developed to facilitate manipulation of the MV, mostly adapted from video-assisted thoracoscopic surgery (VATS), as well as open MV repair techniques.

- Specialized retractors can be used to expose the sub-valvular mitral apparatus.
- The use of GoreTex neochords both as simple chords or loops may offer ease over complex resection techniques.
- Percutaneous knot-tying devices also allow for ease of use.

### 5.6. Anesthesia

Minimally invasive techniques require additional anesthetic considerations in terms of ensuring proper ventilation and monitoring during the entirety of the procedure. A double-lumen tube is typically preferred for single-lung ventilation to access the MV [3,5]. Some will employ an endobronchial blocker or single-lumen ventilation, with early initiation of CPB to facilitate surgery [5]. For cases performed with an aortic endoballoon, bilateral radial artery pressure monitoring is required to detect displacement [3].

Selective intercostal blocks may also be employed to facilitate pain control [3].

## 6. Technical Challenges and Limitations

Learning Curve

￭ There is a learning curve associated with adopting MIMVS. Although visualization and dexterity are superior using the robotic platform, the lack of haptic data requires adjustments in surgical technique. The exact number of procedures required to overcome this curve and attain proficiency is controversial [16].
￭ When establishing a new MIMVS program, a dedicated heart team approach is essential for shared decision making, as well as highly trained personnel (anesthesia, nursing, perfusion).
￭ Case selection is important at the onset of program initiation to minimize CPB and cross-clamp times in the early stages of program and skill development. Patients requiring more complex repairs will be arguably better served with traditional measures or more experienced hands.

Structural Injury

￭ Intra-operative injury of thoracic structures during port placement, especially when deploying the robotic arms.
￭ Lack of tactile feedback poses an increased risk to the surrounding tissues.
￭ Groin complications (seromas, infection and arterial dissections/hematoma) are partially prevented by keeping careful dissection to a minimum, as well as clipping lymphatics [13].
￭ Femoral arterial cannulation increases the risk of retrograde aortic embolization leading to a cerebrovascular event. These potential vascular risks increase with larger cannulas [15].
￭ Phrenic nerve palsy when the pericardium is opened too posteriorly. Early identification and marking of the nerve before incising the pericardium can be helpful. Careful consideration to avoid excessive traction created by the placement of pericardial retraction sutures [11].

Technical Drawbacks

￭ Limited operative field.
￭ Occasional unpredictable difficulty with MV exposure.
￭ Frequent need for a double-lumen endotracheal tube.
￭ Time-consuming. The use of endoscopic instruments and robotic-assisted systems requires more time for set up and manipulation during the procedure.

Instrumentation/Device-dependent

￭ Transthoracic direct aortic clamping carries the risk of aortic, pulmonary artery, left atrial appendage, or left main coronary artery injury [6,12]. The transverse sinus must be well visualized whilst deploying the clamp to avoid catastrophic injuries. This clamp can accidentally be manipulated during the movement of the robotic instruments within the port, causing inadvertent injury to the aorta.
￭ In contrast, an endo-aortic balloon is less stable and prone to dislodging. Associated with a greater risk of aortic dissection and conversion to sternotomy [8].

## 7. Outcomes

Favorable early and late outcomes have been reported for patients who have undergone minimally invasive mitral valve repair. In our own experience involving 75 consecutive patients in 2023 and 2024, we had no operative deaths. Patients had a mean age of 65 ± 12 years. In this selected group, there were no conversions to replacement and no patients had MR > 1+ on early follow-up echocardiography at a mean of 6.2 ± 5.2 months [OHSN REB Protocol 20160395-01H]. Shown below are some results from the published literature (Table 1) [24,25,26,27,28].

Overall, outcomes following minimally invasive mitral valve repair are favorable. There remains, however, some discussion as to whether minimally invasive mitral valve repair is favorable compared to surgical repair performed via sternotomy. Shown below are some studies comparing surgical repair performed via sternotomy versus a less invasive approach (Table 2) [29,30,31,32].

## 8. Conclusions

Minimally invasive and catheter-based techniques will undoubtedly continue their widespread global extension. There is certainly a steep learning curve to be considered, several restrictions posed by the limited operative field along with special considerations pertaining to CPB. Despite the technical drawbacks, MIMVS has the potential to eventually replace median sternotomy as the gold standard surgical approach in most centers regardless of the MV pathology. This is based on proven clinical efficacy, reproducibility, resource utilization and wide capacity for adoption. Patient-driven approaches should not be underestimated, as they are likely to seek a surgeon who can provide the approach they covet. Evidently, meticulous patient selection is critical in pre-determining the results, particularly during the early days/years of the training curve program. Patient safety should remain the number one guiding principle in identifying who would truly benefit from such an approach. A conservative screening algorithm needs to be applied to ensure preferred outcomes. Growing experience and mastery invite more complex challenging repairs with promising outcomes.

## Figures and Tables

**Table 1:** Selected literature describing outcomes following minimally invasive mitral valve surgery.

Study Authors	Year	Number of Patients	Approach	Operative Mortality	Long-Term Outcome
Axtell et al. [25]	2019	101	Small thoracotomy	0%	Freedom from recurrent moderate or severe MR 100%; no reoperation
Feirer et al. [26]	2022	1194	Small thoracotomy	0.6%	Reoperation at 5 years 4.4 ± 3.2%, 10 years 10.3 ± 7.4%, 20 years 16.7 ± 7.4%
Zheng et al. [24]	2022	86 non-robotic 424 robotic	Robotic and non-robotic small thoracotomy	0% for both approaches	5-year freedom from mitral valve reoperation 97.1% following robotic surgery and 95.7% following non-robotic surgery; 3-year freedom from recurrent moderate–severe MR 90% following robotic surgery and 92% following non-robotic surgery
Azcaso et al. [27]	2022	188	Small thoracotomy	1%	MR ≤1+ at discharge in 183 of 188 patients
Polzl et al. [28]	2024	246	Small thoracotomy		>90% survival for this cohort of patients with Barlow valve disease

**Table 2:** Selected literature comparing minimally invasive access versus sternotomy for surgical mitral valve repair.

Authors	Study Year	Number of Patients	Trial Design	Outcomes
Yoo et al. [29]	2014	299 179 minimally invasive 120 sternotomy	Cohort	No 30-day mortality; 5-year freedom from MR >2+ 86.1 ± 4.8% following a minimally invasive approach and 85.3 ± 5.5% in the sternotomy group
Lang et al. [31]	2017	745 501 thoracotomy 244 sternotomy	Cohort	Similar 30-day mortality, 0% in thoracotomy group and 1% in sternotomy group; 5-year survival 93.5 ± 3.7% via thoracotomy and 87.4 ± 3.6% via sternotomy; 5-year reoperation 93.5 ± 2.9% via thoracotomy and 97.9 ± 1.5%
Nasso et al. [32]	2014	80	Randomized controlled trial	Perioperative mortality was 3.75% in both groups; rate of reoperation at 3 years was 2.5% in the minimally invasive group and 1.25% in the sternotomy group
Akowuah et al. [30]	2023	330	Randomized controlled trial	12-week SF-36 results are similar; valve repair rate is 96% in both groups

## Supplementary Materials

The following supporting information can be downloaded at: https://www.mdpi.com/article/10.3390/jcdd11120404/s1, Figure S1: Intraoperative photo denoting surface landmarks used for minimally invasive mitral valve surgery.
