# Supplementary material for: Contemporary Review of Minimally Invasive Mitral Valve Surgery: Current Considerations and Innovations
Source: J Cardiovasc Dev Dis. 2024 Dec 14;11(12):404. doi: 10.3390/jcdd11120404 (PMC11677698; doi:10.3390/jcdd11120404)
Supplement: Supplementary file 1 [file jcdd-11-00404-s001.zip › jcdd-3311387-supplementary.pdf]

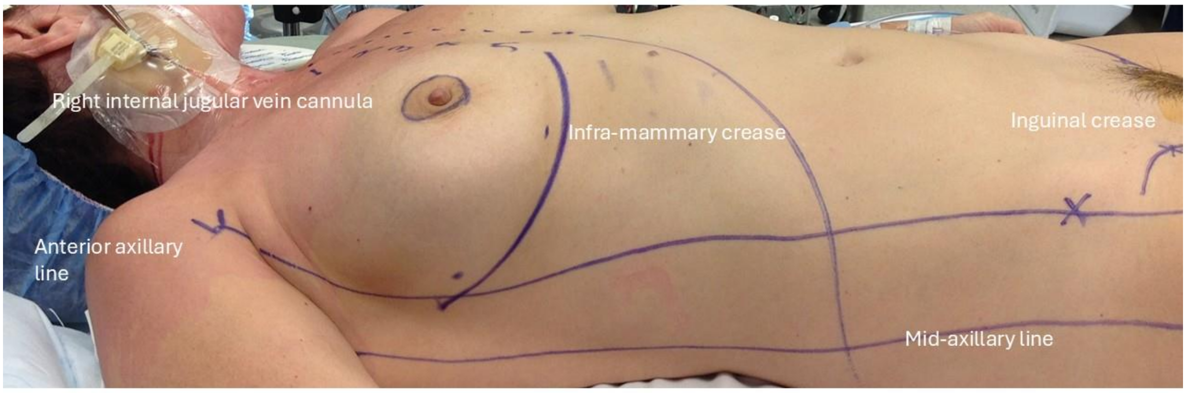

Figure S1. Intraoperative photo denoting surface landmarks used for minimally invasive mitral valve surgery
